# 764 Ain’t no sunshine when she’s burnt - An empowering approach to improving sunburn prevention

**DOI:** 10.1093/jbcr/irac012.317

**Published:** 2022-03-23

**Authors:** Bartlomiej Bednarz, Mehul Thakkar, Timothy Schrire, Sankhya Sen

**Affiliations:** North Bristol NHS Trust, Bristol, England; Glasgow Royal Infirmary, London, England; North Bristol NHS Trust, Bristol, England; North Bristol NHS Trust, Bristol, England

## Abstract

**Introduction:**

Outdoor activities and the associated sun exposure is a mainstay of summer activities for many children. This UV exposure presents a significant risk to children of Anglo-Saxon or Celtic origin. Whilst UV exposure is important for mood and vitamin D synthesis, too much results in premature ageing and increased risk of skin malignancy. We propose a classification system of these burns according to pattern of injury, related directly to patient age.

**Methods:**

We collated data concerning paediatric sun burns over a five year period (2015-2019). This was collected from our Regional Burns Unit, covering a population of roughly 5 million. We present an analysis of age, sex, total body surface area and location of the burn, correlated with the local UV index on the day of injury.

**Results:**

We identified 138 patients presenting with sunburn. They were predominantly (71%) in males, with an age range of 1m to 16y (average 7y9m median 6y10m). All burns were superficial partial thickness in nature and averaged 2.2% TBSA (range 0.1%- 15%). No patients required surgery, however, 19 required admission for analgesia (4 of whom stayed >1 day). All burns occurred between the months of April and September, with 24 (17%) associated with foreign travel. The average UV index, where applicable, was 6.5 (ranging from 3.6 to 8). The majority of patients had no or little sun protection applied.

We propose three patterns of sunburn from analysing the data:

Neonatal and toddler sunburns usually happen in the presence of a caregiver, but with inadequate protection, such as muslin cloth or a poorly shaded area. These injuries, whilst typically small, have significant impact, as radiation injuries are cumulative and may affect future skin cancer risk for young patients.

Young (4-9 yo) children get sun burn whilst out with their friends or in the garden with parents. Inadequate sun protection or prolonged exposure is a key preventable factor.

Teenagers (12-16) are usually sun burnt whilst on a foreign trip to warmer countries or whilst with their friends during outdoor activities such as waterparks or surfing. Again minimal or no sunscreen application is a preventable cause.

**Conclusions:**

In conclusion, this classification system, we seek to inform presenting units with a guide to management and common patterns, therefore improving the sun protection for children. Applicability of Research to Practice:

We identify three preventable patterns of sunburns in paediatric population. This allows parents and health professionals to be educated in a targeted way on prevention of injuries which may have a lifelong effect on skin cancer malignancies risk.

